# Risk of cardiovascular events from current, recent, and cumulative exposure to abacavir among persons living with HIV who were receiving antiretroviral therapy in the United States: a cohort study

**DOI:** 10.1186/s12879-017-2808-8

**Published:** 2017-10-27

**Authors:** Kunchok Dorjee, Sanjiv M. Baxi, Arthur L. Reingold, Alan Hubbard

**Affiliations:** 1 Division of Epidemiology, School of Public Health, University of California Berkeley, Hall Berkeley, 101 Haviland, CA 94720-7358 USA; 2 Department of Medicine, University of California San Francisco, San Francisco, California, USA; 3 Division of Biostatistics, School of Public Health, University of California Berkeley, Berkeley, California, USA; 4 Division of Infectious Diseases, School of Medicine, Johns Hopkins University, Baltimore, MD USA

**Keywords:** HIV, Abacavir, Anti-retroviral therapy, Cardiovascular disease

## Abstract

**Background:**

There is ongoing controversy regarding abacavir use in the treatment of HIV infection and the risk of subsequent development of cardiovascular disease. It is unclear how the risk varies as exposure accumulates.

**Methods:**

Using an administrative health-plan dataset, risk of cardiovascular disease events (CVDe), defined as the first episode of an acute myocardial infarction or a coronary intervention procedure, associated with abacavir exposure was assessed among HIV-infected individuals receiving antiretroviral therapy across the U.S. from October 2009 through December 2014. The data were longitudinal, and analyzed using marginal structural models.

**Results:**

Over 114,470 person-years (*n* = 72,733) of ART exposure, 714 CVDe occurred at an incidence rate (IR) (95% CI) of 6·23 (5·80, 6·71)/1000 person-years. Individuals exposed to abacavir had a higher IR of CVDe of 9·74 (8·24, 11·52)/1000 person-years as compared to 5·75 (5·30, 6·24)/1000 person-years for those exposed to other antiretroviral agents. The hazard (HR; 95% CI) of CVDe was increased for current (1·43; 1·18, 1·73), recent (1·41; 1·16, 1·70), and cumulative [(1·18; 1·06, 1·31) per year] exposure to abacavir. The risk for cumulative exposure followed a bell-shaped dose-response curve peaking at 24-months of exposure. Risk was similarly elevated among participants free of pre-existing heart disease or history of illicit substance use at baseline.

**Conclusion:**

Current, recent, and cumulative use of abacavir was associated with an increased risk of CVDe. The findings were consistent irrespective of underlying cardiovascular risk factors.

**Electronic supplementary material:**

The online version of this article (10.1186/s12879-017-2808-8) contains supplementary material, which is available to authorized users.

## Background

Cardiovascular disease (CVD) accounts for approximately 16% of deaths among persons living with HIV (PLWH) [1]. Risk factors for CVD are more prevalent among PLWH [2], and use of various antiretroviral (ARV) drugs has been shown to be associated with an increased risk of CVD [3]. With rapid expansion of antiretroviral therapy (ART) coverage both domestically and abroad, researchers and clinicians have become increasingly aware of potential ARV drug-related adverse events. Whether the commonly used ARV drug abacavir is associated with an increased risk of CVD has been intensely debated. Abacavir sulfate is a guanosine analog nucleoside reverse transcriptase inhibitor that possesses retroviral suppressive properties similar to tenofovir [4], and is a commonly prescribed backbone ARV agent. However, the writing of prescriptions of abacavir declined after the Data Collection on Adverse Events of Anti-HIV Drugs (D:A:D) study group reported in 2008 an increased risk of acute myocardial infarction (AMI) among PLWH exposed to abacavir [5–7]. Independent investigations that were subsequently carried out have both supported [7–17] and refuted [18–23] the D:A:D study group’s findings.

While studies conducted more recently have generally suggested an increased risk of CVD from abacavir exposure [8, 10, 14, 17], they were limited by few outcomes, with results occasionally underpowered [8, 17]. Failure to identify a clear underlying biological mechanism to explain the epidemiologic findings has added to the deliberation [24]. Furthermore, there has also been a lack of consensus regarding whether the risk of CVD from exposure to abacavir reverses within a few months of stopping the drug [5, 16] and a lack of understanding on how the risk varies as exposure accumulates. In this study, we have sought to address these limitations by investigating the risk of CVD events (CVDe) from current, recent, and cumulative exposure to abacavir among PLWH using conventional and causal statistical methods.

## Methods

### Study design, sample collection and participants

**Fig. 1:**
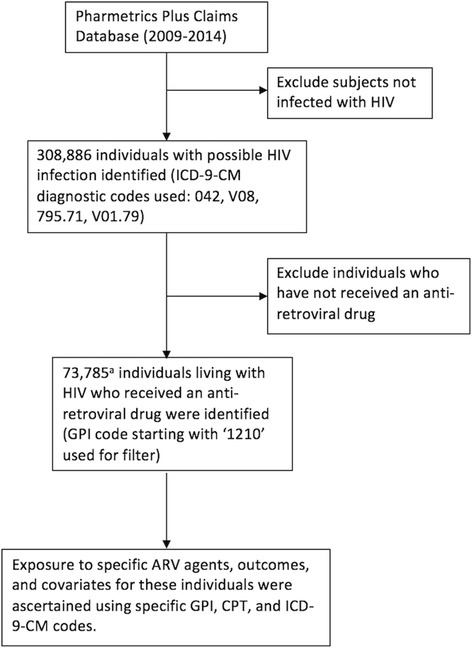
Algorithm for defining the study cohort from the IMS’ PharMetrics Plus claims database. GPI: generic product identifier; CPT: current procedural terminology; ICD-9-CM: International Classification of Disease, 9th Revision, Clinical Modification. ^a^Additional filter (age ≥ 18) applied to obtain final cohort

The risk of CVDe was assessed among PLWH who started ARV drugs in the U.S. between October 1, 2009 and December 31, 2014. Data were obtained from medical and prescription claims data included in the IMS’ PharMetrics Plus database. October 1, 2009 was the earliest possible date for complete availability of relevant data; ART prescription history prior to this date was not available. PharMetrics Plus is a large health plan insurance claims database in the U.S., and is comprised of adjudicated claims for more than 150 million unique enrollees from across four regions of the U.S. [25]. The data undergo a series of quality checks to minimize errors. This study used a pre-defined algorithm (Fig. 1) to extract and define the study population of PLWH exposed to any ART in the database. The study population was restricted to those ≥18 years of age. The baseline time point was defined as the date of ART initiation in the database and individual follow up time was censored at the first of three events after baseline: 1) first occurrence of CVDe, 2) last recorded date of ART receipt, 3) December 31, 2014. The study was approved by the Committee for Protection of Human Subjects at the University of California, Berkeley.

### Exposure, covariate, and outcome definitions

Exposures to specific ARV agents were identified by generic product identifier (GPI) codes. Person-time of exposure to abacavir was compared to exposure to ARV agents other than abacavir. Any two prescriptions for an ARV agent separated by <30 days were combined to represent a single continuous exposure; gaps ≥30 days were not combined and this person-time was not included in the analysis. These data are longitudinal, and each subject’s follow up time was divided into consecutive one-month periods during which treatment was allowed to vary. The values of covariates were updated at the start of each month. The outcome of CVDe for an individual was defined as the first occurrence of an AMI or receipt of a coronary artery intervention procedure (i.e. percutaneous coronary intervention or coronary artery bypass graft) after baseline. AMI and coronary artery intervention procedures were ascertained using the International Classification of Disease, 9th Revision, Clinical Modification (ICD-9-CM) or Current Procedural Terminology (CPT) codes, respectively (Additional file 1: Table S1). The ICD-9 code used for AMI (410.xx) has been previously validated in another claims database [26].

The temporal ordering of covariate, treatment, and outcome allowed for a time-varying analysis, and the opportunity for causal interpretations. The first observation of a time-dependent covariate corresponded to its baseline value and once a health condition developed, an individual was assumed to have the condition for the remainder of the study. Current exposure to abacavir was defined as exposure (yes/no) during each one-month observation period. Recent exposure was defined as exposure (yes/no) in the previous six months (inclusive of the current month). Cumulative exposure was defined as the total duration of exposure an individual had received at a particular time point in one-month increments, updated monthly. Duration of exposure ceased to accumulate upon discontinuation of the drug but resumed if the drug was restarted. HIV-infection status and covariates were ascertained using the ICD-9-CM or CPT codes (Additional file 1: Table S1).

### Statistical analysis

The risk of CVDe from a current, recent, and cumulative exposure to abacavir was estimated by the parameters of pooled logistic regression marginal structural models using stabilized inverse probability of treatment weights (sIPTW) [27]. The sIPTW was generated from four treatment models – two each for the numerator and the denominator of the weight [16]. For the denominator, the time point specific probability of exposure initiation was first estimated by fitting a main term pooled logistic regression to data up to the individual’s first month of receiving the exposure or end of follow up for those who were never exposed. The probability of exposure continuation was then estimated by fitting the model to data after the first month of starting the exposure. The denominator was modelled as a function of baseline covariates: gender, tobacco use/smoking (ever), substance or alcohol abuse (ever), serologic evidence of hepatitis B and C infections, history of stroke, cancer or old myocardial infarction, and time-dependent covariates: age, year of ART initiation, body weight, receipt of hypoglycemic agents (i.e. sulfonylureas, biguanides, insulin, thiazolidinedione) or medications for CVDe (i.e. aspirin, beta-blocker, angiotensin converting enzyme inhibitor, angiotensin receptor blocker, calcium channel blocker, statins) or diagnoses of: chronic kidney disease (CKD), dyslipidemia, heart failure, cardiac dysrhythmia, atherosclerosis, diabetes mellitus, and hypertension. The exposure continuation model additionally contained a variable for past month’s exposure status. The probabilities for the numerator of the sIPTW were similarly modelled but as a function of baseline covariates only. The follow-up time was modeled as a function of natural cubic splines with three internal knots placed at 25th, 50th and 75th percentiles. The marginal structural model was adjusted for the sIPTW and the baseline covariates. Same treatment weights were used for estimation of CVDe risk from current, recent, and cumulative exposure to abacavir. In order to assess the change in risk over time, the adjusted and marginal models were fit as a function of categories of cumulative exposure, i.e., never exposed, 1–6, 7–12, 13–18, 19–24, and >25 months of exposure. In sensitivity analyses, the study population was restricted to individuals free of CVD at baseline, and to individuals without a history of alcohol and substance abuse at baseline. Sensitivity analyses were additionally carried out to assess if the risk of CVDe from abacavir exposure differed after adjusting for other anti-retroviral agents. Using the same sIPTW models, we tested for interaction to see whether risk of CVDe from current abacavir exposure is modified in the presence of 13 different risk factors (Additional file 1: Table S5). In addition to the marginal structural results, corresponding results from unadjusted and adjusted Cox models were calculated. This study assumes uninformative censoring. Data were extracted and processed from the main claims databases using TERADATA (Dayton, OH), SAS version 9.1 (SAS Institute, Cary, NC), and STATA version 13.1 (StataCorp, College Station, TX). The marginal structural models were implemented in STATA version 13.1, based on Fewell et al. [28]. The rationale, definition, and implementation of the marginal structural models are described in Additional file 1: Appendix 1.

## Results

### Study population and incidence rates

There were 72,733 participants contributing 114,470 person-years of exposure to antiretroviral agents. On average, participants were exposed to ART for 1.5 years. The mean age of the study population was 46 years and 82% were males. The characteristics of the study population at baseline and summary of exposure to various antiretroviral drugs are described in Tables 1, 2 respectively. Overall, 714 CVDe occurred at an incidence rate of 6.23 (95% CI: 5.80, 6.71)/1000 person-years. Of the 714 outcomes, 137 were observed over 14,060 person-years of current exposure to abacavir at an incidence rate of 9.74 (95% CI: 8.24, 11.52)/1000 person-years, as compared to 577 outcomes over 100,410 person-years with an incidence rate of 5.75 (95% CI: 5.30, 6.24)/1000 person-years for those currently exposed to other ARV drugs. The incidence rate was highest for those exposed to abacavir between 13 and 18 months (11.32/1000 person-years) (Table 3). Of the 714 CVDe, 548 were cases of AMI. The overall incidence rate of AMI was 4.78 (95% CI: 4.39, 5.19)/1000 person-years (Additional file 1: Table S2). We calculated a population attributable risk (PAR) associated with abacavir exposure as:

$$ {\displaystyle \begin{array}{l}{\left(\frac{\mathrm{Risk}\  \mathrm{of}\  \mathrm{CVDe}\  \mathrm{in}\  \mathrm{Total}\  \mathrm{Population}\hbox{-} \mathrm{Risk}\  \mathrm{of}\  \mathrm{CVDe}\  \mathrm{in}\  \mathrm{Unexposed}\  \mathrm{Population}}{\mathrm{Risk}\  \mathrm{of}\  \mathrm{CVDe}\  \mathrm{in}\  \mathrm{Total}\  \mathrm{Population}}\right)}^{\ast }100\\ {}={\left(\frac{\left(6.23/1000\right)\hbox{-} 5.75/1000}{\left(6.23/1000\right)}\right)}^{\ast }100=8\%.\end{array}} $$

**Table 1:** Baseline characteristics of persons living with HIV in the US receiving antiretroviral agents, stratified by exposure to abacavir, in the IMS PharMetric Plus claims database from October 1, 2009 through December 31, 2014

Characteristic	Exposed to abacavir (*n* = 8530)	Exposed to other ARV agents (reference group) (*n* = 64,203)
Age, median (IQR)	48 (43–54)	46 (39–52)
Male	6889 (80.76)	52,402 (81.62)
Region		
East	2057 (24.11)	15,336 (23.89)
Mid-West	1370 (16.06)	12,104 (18.85)
South	3986 (46.73)	29,179 (45.45)
West	1117 (13.09)	7584 (11.81)
Year of ART initiation in the database		
2009	3590 (42.09)	20,440 (31.84)
2010	1120 (13.13)	8578 (13.36)
2011	1147 (13.45)	9121 (14.21)
2012	801 (9.39)	7824 (12.19)
2013	643 (7.54)	7259 (11.39)
2014	1229 (14.41)	10,981 (17.10)
Ever substance abuse	1290 (15.12)	11,837 (18.44)
Ever alcohol abuse	273 (3.20)	2750 (4.28)
Ever tobacco use/smoking	1198 (14.04)	10,385 (16.18)
Body mass index > 24.9	116 (1.36)	1130 (1.76)
Essential hypertension	766 (8.98)	5026 (7.83)
Diabetes mellitus	366 (4.29)	2049 (3.19)
Chronic Kidney Disease	265 (3.11)	492 (0.77)
Dyslipidemia	820 (9.61)	5552 (8.65)
Lipodystrophy	36 (0.42)	129 (0.20)
Pre-existing heart disease^a^	242 (2.84)	1768 (2.75)
Receipt of medications for heart disease^b^	819 (9.60)	4816 (7.50)
History of stroke	25 (0.29)	160 (0.25)
Symptomatic HIV disease	2313 (27.12)	18,839 (29.34)
Hepatitis B	69 (0.81)	612 (0.95)
Hepatitis C	141 (1.65)	896 (1.40)
History of cancer	438 (5.13)	4152 (6.47)

All reported as N (%) unless otherwise stated

^a^Prior myocardial infarction, heart failure, cardiac dysrhythmias, and atherosclerosis

^b^Aspirin, beta-blocker, statins, angiotensin converting enzyme inhibitor, angiotensin receptor blocker, calcium channel blocker

**Table 2:** Summary of exposure to various antiretroviral drugs among people living with HIV in the US in the IMS Pharmetrics Plus Claims database stratified by regimens containing and not containing abacavir from October 1, 2009 through December 31, 2014

Antiretroviral drug	Total exposure	Exposure in antiretroviral regimens containing abacavir	Exposure in antiretroviral regimens not containing abacavir
Tenofovir			
Persons with any exposure – n (%)	55,804 (76.7)	Not calculated	Not calculated
Total person-years of exposure	80,939	2528	78,410
Cumulative exposure (years) per person – mean	2.1	0.6	2.3
Lamivudine			
Persons with any exposure – no. (%)	14,106 (19.4)	Not calculated	Not calculated
Total person-years of exposure	19,886	10,736	9150
Cumulative exposure (years) per person – mean	0.5	2.2	0.3
Zidovudine			
Persons with any exposure – n (%)	6883	Not calculated	Not calculated
Total person-years of exposure	9665	2074	7591
Cumulative exposure (years) per person – mean	0.3	0.5	0.2
Emtricitabine			
Persons with any exposure – n (%)	53,377 (73.4)	Not calculated	Not calculated
Total person-years of exposure	76,466	693	75,774
Cumulative exposure (years) per person – mean	1.9	0.2	2.2
Efavirenz			
Persons with any exposure – n (%)	29,795	Not calculated	Not calculated
Total person-years of exposure	45,930	2466	43,464
Cumulative exposure (years) per person – mean	1.2	0.6	1.3
Nevirapine			
Persons with any exposure – n (%)	3879	Not calculated	Not calculated
Total person-years of exposure	5880	1136	4744
Cumulative exposure (years) per person – mean	0.1	0.04	0.1
Rilpivirine			
Persons with any exposure – n (%)	4345	Not calculated	Not calculated
Total person-years of exposure	3778	103	3675
Cumulative exposure (years) per person – mean	0 (0–0)	0 (0–0)	0 (0–0)
Atazanavir			
Persons with any exposure – n (%)	10,470	Not calculated	Not calculated
Total person-years of exposure	13,862	3026	10,836
Cumulative exposure (years) per person – mean	0.4	0.6	0.3
Darunavir			
Persons with any exposure – n (%)	8871	Not calculated	Not calculated
Total person-years of exposure	10,394	1310	9084
Cumulative exposure (years) per person – mean	0.3	0.3	0.3
Lopinavir			
Persons with any exposure – n (%)	5596	Not calculated	Not calculated
Total person-years of exposure	7150	1230	5920
Cumulative exposure (years) per person – mean	0.2	0.3	0.2
Fosamprenavir			
Persons with any exposure – n (%)	1964	Not calculated	Not calculated
Total person-years of exposure	2699	698	2001
Cumulative exposure (years) per person – mean	0.1	0.2	0.1
Raltegravir			
Persons with any exposure – n (%)	10,537	Not calculated	Not calculated
Total person-years of exposure	13,663	1731	11,932
Cumulative exposure (years) per person – mean	0.4	0.4	0.4

**Table 3:** Incidence rate (IR) of cardiovascular disease events^a^ (CVDe) among persons living with HIV exposed to abacavir for various durations

Duration of exposure to abacavir (months)	Person-years	No. of CVDe	Incidence rate per 1000 people (95% CI)
Never exposed	98,857	561	5.68 (5.22, 6.16)
1–6	4757	51	10.72 (8.15, 14.11)
7–12	3125	31	9.92 (6.98, 14.11)
13–18	2208	25	11.32 (7.65, 16.76)
19–24	1663	18	10.82 (6.82, 17.18)
>25	3860	28	7.25 (5.01, 10.51)

^a^Includes acute myocardial infarction and coronary intervention procedures

### Factors associated with Abacavir use

At baseline, abacavir recipients had a higher prevalence of essential hypertension, diabetes mellitus, chronic kidney disease (CKD), dyslipidemia, lipodystrophy, heart disease, and use of cardiovascular medications (Table 1). In the pooled logistic regression model, older age, a diagnosis of CKD, symptomatic HIV infection, and presence of lipodystrophy were associated with an increased probability of receiving abacavir (Additional file 1: Table S3).

### Predictors of outcome

**Fig. 2:**
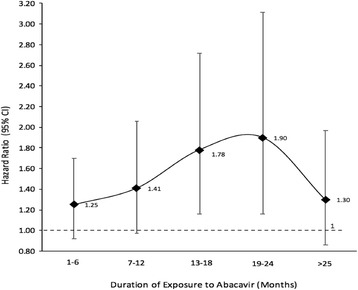
Risk of cardiovascular disease events associated with increasing durations of exposure to abacavir as compared to those never exposed. See Table 3 and S4 table for covariate adjustment

The sIPTW models showed the risk of CVDe (HR; 95% CI) was increased for current (1.43; 1.18, 1.73), recent (1.41; 1.16, 1.70) and cumulative (1.18; 1.06, 1.31) exposure (per year) to abacavir (Table 4). Separate models were run for each of current, recent, and cumulative exposure. The unadjusted and adjusted Cox models also showed increased risk for these exposures (Table 4). On further assessment of the risk from cumulative exposure, the HR varied with the duration of exposure in an inverted U-shaped pattern (Table 5 and Fig. 2 **)**; the relative hazard continued to increase up to 24 months of exposure, after which it decreased to non-significant levels but remained elevated compared to those never exposed to abacavir. Older age, male sex, tobacco use, other heart diseases, prior AMI, use of CVD-related medications, diabetes mellitus, and dyslipidemia were each associated with increased hazard of CVDe in the adjusted Cox model (Additional file 1: Table S4). We also assessed whether the risk was reversible after six months of stopping abacavir by comparing those with any abacavir exposure prior to but not in the last six months including the current month to those never exposed and found that the risk (HR; 95% CI) remained elevated (sIPTW model: 1.69; 0.89, 3.20; adjusted Cox model: 2.08; 1.17, 3.71). In tests of interactions, we observed that the risk of CVDe associated with abacavir use was more pronounced for age < 45 years (interaction *p*-value: 0.028) and for people without prior heart disease (interaction *p*-value: 0.016) (Additional file 1: Table S5).

**Table 4:** Risk of cardiovascular disease events associated with current, recent, and cumulative exposure to abacavir among persons living with HIV, in the IMS PharMetric Plus claims database from October 1, 2009 through December 31, 2014

Exposure^a^	Unadjusted Cox Model HR (95% CI; *p* value)	Adjusted Cox Model HR^b^ (95% CI; *p* value)	Marginal Structural Model (sIPTW) HR^c^ (95% CI; p value)
Current^d^	1.70 (1.41, 2.05; *p* < 0.001)	1.32 (1.09, 1.60; *p* = 0.004)	1.43 (1.18, 1.73; *p* = 0.001)
Recent^e^	1.66 (1.38, 2.00; *p* = 0.001)	1.28 (1.06, 1.54; *p* = 0.01)	1.40 (1.16, 1.69; *p* = 0.001)
Cumulative (per year)	1.24 (1.12, 1.37; *p* < 0.001)	1.13 (1.02, 1.25; *p* = 0.02)	1.18 (1.06, 1.31; *p* = 0.002)

^a^Separate models run for each of current, recent, and cumulative exposure to abacavir

^b^Adjusted for baseline covariates: gender, tobacco use (ever), substances or alcohol abuse (ever), symptomatic HIV disease, serologic evidence of hepatitis B & C infections, history of stroke, history of cancer, prior myocardial infarction, and time-dependent covariates: age, calendar year, body weight, receipt of anti-hyperglycemic agents (sulfonylureas, biguanides, insulin, thiazolidinedione), receipt of medications for heart disease (i.e. aspirin, beta-blocker, angiotensin converting enzyme inhibitor, angiotensin receptor blocker, calcium channel blocker), and diagnoses of: diabetes mellitus, chronic kidney disease, dyslipidemia, heart failure, cardiac dysrhythmia, atherosclerosis, and hypertension

^c^In addition to adjusting for weights generated from the treatment model using the time-fixed and time-dependent covariates in the adjusted Cox model, the marginal models are adjusted for time-fixed/baseline covariates: sex, ever tobacco use, ever alcohol or substance abuse, symptomatic HIV disease, serologic evidence of hepatitis B & C infections, history of stroke, history of cancer, prior myocardial infarction, and baseline values of time-dependent covariates: age, calendar year, receipt of anti-hyperglycemic agents, receipt of medications for heart disease, and diagnoses of: diabetes mellitus, chronic kidney disease, dyslipidemia, heart failure, cardiac dysrhythmia, atherosclerosis, and hypertension

^d^Referent group is those not currently exposed to abacavir

^e^Referent group is those not recently exposed to abacavir

**Table 5:** Risk of cardiovascular disease among HIV-infected individuals exposed to abacavir for various durations

Duration of exposure (months)	HR (95% CI; *p* value) Unadjusted Cox Model	Adjusted Cox Model HR^a^ (95% CI; *p* value)	Marginal Structural Model HR^b^ (95% CI; *p* value)
Never exposed	Referent	Referent	Referent
1–6	1.66 (1.23, 2.25; *p* = 0.001)	1.24 (0.92, 1.67; *p* = 0.163)	1.25 (0.92, 1.70; *p* = 0.150)
7–12	1.69 (1.15, 2.47; *p* = 0.007)	1.27 (0.87, 1.86; *p* = 0.219)	1.41 (0.97, 2.06; *p* = 0.073)
13–18	2.28 (1.48, 3.54; *p* < 0.001)	1.71 (1.10, 2.65; *p* = 0.016)	1.78 (1.16, 2.72; *p* = 0.009)
19–24	2.09 (1.26, 3.47; *p* = 0.004)	1.62 (0.98, 2.69; *p* = 0.060)	1.90 (1.16, 3.11; *p* = 0.011)
>25	1.45 (0.97, 2.18; *p* = 0.071)	1.20 (0.80, 1.80; *p* = 0.386)	1.30 (0.86, 1.97; *p* = 0.208)

^a^Adjusted for baseline covariates: gender, tobacco use (ever), substances or alcohol abuse (ever), symptomatic HIV disease, serologic evidence of hepatitis B & C infections, history of stroke, history of cancer, prior myocardial infarction, and time-dependent covariates: age, calendar year, body weight, receipt of anti-hyperglycemic agents, receipt of medications for heart disease, and diagnoses of: diabetes mellitus, chronic kidney disease, dyslipidemia, heart failure, cardiac dysrhythmia, atherosclerosis, and hypertension

^b^In addition to adjusting for weights generated from the treatment model using all the time-fixed and time-dependent covariates in the adjusted Cox model, the marginal model is adjusted for time-fixed/baseline covariates: sex, ever tobacco use, ever alcohol or substance abuse, symptomatic HIV disease, serologic evidence of hepatitis B & C infections, history of stroke, history of cancer, prior myocardial infarction, and baseline values of time-dependent covariates: age, calendar year, receipt of anti-hyperglycemic agents, receipt of medications for heart disease, and diagnoses of: diabetes mellitus, chronic kidney disease, dyslipidemia, heart failure, cardiac dysrhythmia, atherosclerosis, and hypertension

### Sensitivity analyses

In a sensitivity analysis, we observed a 53% higher risk of CVDe (sIPTW model) for current exposure to abacavir among individuals without a prior AMI or heart disease at baseline (Additional file 1: Table S6). This relationship was also assessed by excluding other heart diseases (heart failure, cardiac arrhythmia, atherosclerosis, or receipt of cardiovascular medications) from the adjustment set of covariates for both the marginal and the adjusted Cox model, with similar results. The risk also remained elevated by 41% when the study population was restricted to individuals not using illicit substances or alcohol at baseline (Additional file 1: Table S6). We further tested for CVDe risk from abacavir use after adjusting for cumulative exposure to other antiretroviral agents (tenofovir, emtricitabine, zidovudine, lamivudine, lopinavir, atazanavir, darunavir, efavirenz, nevirapine, rilpivirine, and raltegravir) using sIPTW models and found elevated risk (HR; 95% CI) for current (1.38; 1.12, 1.68), recent (1.34; 1.09, 1.64), and cumulative exposure (1.16; 1.03, 1.31). We then replicated the D:A:D model [5] for cumulative exposure by including recent exposure in the same model as cumulative exposure and observed that although our hazard ratio estimate for risk from cumulative exposure (per year) remained elevated (HR:1.08; 95% CI: 0.89, 1.30), it decreased to a non-statistically significant level. When we modelled the risk by partitioning the cumulative exposure into various durations, we observed a similar increased risk [HR (95% CI)] pattern as observed in our primary analysis (Table 4): 1–6 months: 1.91 (0.95–3.83); 7–12 months: 2.58 (1.16–5.71); 13–18 months: 2.68 (1.17–6.11); 19–24 months: 2.90 (1.37–6.17); and ≥25 months: 2.13 (0.93–4.88).

## Discussion

In a large database claims-based study, we found an increased risk of CVDe associated with exposure to current, recent, and cumulative exposure to abacavir using both adjusted Cox and marginal structural models estimated with inverse probability treatment weights. The overall incidence rate of AMI in this study was 4.78/1000 person-years, which compares to 3.3/1000 person-years in the 2008 D:A:D study. AMI incidences of 1.41/1000 people and 1.2/1000 people were seen in the general population in Olmstead county in Minnesota in 2006 and in men 35–65 years of age in the Framingham study population, respectively [29, 30]. This relatively higher incidence of AMI in the PLWH could be due to HIV infection [31–33], ART use [3], or both; PLWH have been shown to have more risk factors for CVD as compared to the general population [31–33]. The incidence rates of AMI associated with exposure to abacavir in this study (6.9/1000 person-years) and in the D:A:D study (6.1/1000 person-years) were ~4–5 fold higher than the general population estimates and approximately 2-fold higher than in the general population of PLWH [2, 31–34]. Some of the difference in results between this study and the D:A:D study including higher incidence rate of AMI in this study could be because participants in this study were all exposed to ART whereas the D:A:D study included individuals who had not yet started ART, as well as those who had discontinued ART totally. We calculated a population attributable risk of 8%. This means 8% (*n* = 57) of the total CVDe risk in the PLWH could be prevented if abacavir was not used, assuming a causal relationship between abacavir use and CVDe risk.

In an attempt to characterize an underlying biological mechanism for the increase in CVDe risk associated with abacavir use, we assessed how the risk varied with duration of exposure. The relative hazard of AMI increased with increasing duration of exposure in an inverted U-shaped pattern, peaking between 13 and 24 months of exposure and leveling off thereafter, suggesting a dose response relationship between cumulative time exposed to abacavir and risk, up to 24 months. This result agrees with earlier finding by Young et al. in which they first showed that the risk of CVD increased with increasing duration of exposure, with greatest risk between 6 and 36 months and exposure beyond 36 months adding little to the risk, suggesting a dose-response pattern. We observed the dose-response relationship for various durations of cumulative exposure after controlling for recent exposure as well in addition to other variables in the model; the D:A:D study group [5] had reported that the observed risk for cumulative exposure disappeared after adjusting for recent exposure, meaning that the CVD risk existed only up to first 6 months of exposure, after which the risk reversed. In a separate model, we tested the risk reversibility and found a 69% increased risk of CVDe among those who had stopped abacavir prior to last six months, suggesting a risk not reversible within six months of stopping the drug. We did not formally test whether the inverted U-shaped curve described for cumulative exposure provides a better fit to the observed risk estimates than a simple linear association.

Whereas this and Young et al.’s study results do not support an underlying mechanism related to immediate exposure to abacavir, the results are not consistent with an atherogenic mechanism, in which an ongoing or increasing risk would be expected with an increasing duration of exposure, without necessarily reaching a peak effect and leveling off after 24 months. The finding of an early peak in the increased risk of AMI in the course of abacavir treatment is helpful in understanding how risk may change with continuing versus changing therapy. The study results presented here suggest a reversible but more gradual underlying mechanism with a longer lasting impact that regresses more slowly after removal of the exposure.

Prior work has suggested that abacavir-induced platelet hyper-reactivity and aggregation could potentially lead to thrombosis and myocardial infarction [35–37]. Specifically, abacavir may induce platelet hyper-reactivity by competitive inhibition of a nitric oxide-induced soluble guanylyl cyclase via its active metabolite, carbovir-triphosphate, leading to a decreased production of cyclic guanosine monophosphate, an inhibitor of platelet aggregation and secretion [24, 35, 36, 38]. It is possible that abacavir may trigger an acute platelet response leading to endothelial injury with a longer lasting impact. It is also unclear whether abacavir may exert its effect on CVD risk through an increase in inflammatory biomarkers. While the SMART/INSIGHT study investigators [15], Kristoffersen et al. [39], and Hileman et al. [40] showed evidence for a possible role of inflammatory biomarkers in causing CVD among abacavir users [e.g. increased levels of high sensitivity c-reactive protein (hsCRP) and interleukin-6], several other studies have shown that levels of inflammatory biomarkers such as hsCRP, interleukin-6, selectin P and E, D-dimer, vascular adhesion molecule-1, intercellular adhesion molecule-1, and tumor necrosis factor alpha are not elevated after exposure to abacavir [41–54]. Future interdisciplinary studies may explore these areas by bridging basic, translational and clinical science to provide additional insights into the mechanisms underlying abacavir-associated cardiovascular risk. We have not established a clear reason for observing a higher risk of CVDe associated with abacavir use among the younger age-group and individuals without a pre-existing cardiac condition in the test of interactions. While we acknowledge the exploratory nature of the analyses for interaction testing with the possibility that the results could be due to chance, the observation of a higher CVDe risk in individuals without prior heart disease may stand to support the finding of an increased risk in younger age people. The increased CVDe risk in younger age people could also reflect a higher prevalence of cocaine and injection drug use among them [19]. It would be important to test in other populations whether CVD risk associated with abacavir use differs by age.

We used the sIPTW approach because individuals with certain risk factors for CVD such as CKD, hypertension, diabetes mellitus, and dyslipidemia, may be preferentially channeled into (or away from) receiving abacavir based on its known toxicity in the presence of these conditions. The sIPTW approach may also be necessary because post-baseline values of these variables may simultaneously serve as confounders and causal intermediates; adjusting for these through traditional methods can lead to biased results [55]. Under such settings, the use of inverse probability weights provides a valuable tool for balancing confounders across exposure groups without conditioning on variables affected by treatment [55, 56]. Some of our results for various durations of cumulative exposure appreciably differed between conventional Cox models and marginal structural models. For example, the hazard ratios (95% CI, *p* value) for 7–12 months, 13–18 months, and 19–24 months of cumulative exposure were 1.27 (0.87–1.86; *p* = 0.219), 1.71 (1.10, 2.65; *p* = 0.016), and 1.62 (0.98, 2.69; *p* = 0.060), respectively, in adjusted Cox models. The corresponding hazard ratios from marginal structural models were 1.41 (0.97, 2.06; *p* = 0.073), 1.78 (1.16, 2.72; *p* = 0.009), and 1.90 (1.16, 3.11; *p* = 0.011) (Table 4).

A key strength of this study is the application of conventional and robust methods to address key study questions while using a very large U.S. health-plan dataset containing longitudinal information on usage of ART in >70,000 PLWH receiving care across the U.S. The recency of the data is an asset. Most studies that showed an association between abacavir use and CVD risk so far were hospital based [5, 9, 10, 14, 16–19] and hence may be subject to similar bias, such as channeling bias, that could arise from specific prescription behavior of physicians. Therefore, reproduction of the results in another representative population, such as that enrolled in the claims database, would be relevant and important. The similarity of these results to those from prior studies, the reproducibility of the results in the sensitivity analyses, and the finding of a background incidence rate of AMI comparable to that found in other studies are reassuring. A limitation of the study is that the ICD-9 and CPT diagnostic codes used may be prone to coding errors; however, such errors are likely to affect the exposure groups non-differentially and may not bias the study results. It is possible that information on covariates, such as body-weight, for which re-imbursement may not be sought could be under-reported in the database. Again, we expect this problem to exist non-differentially across exposure groups. This is an observational cohort study and is therefore subject to confounding from unmeasured factors and possible channeling bias; we have attempted to account for the latter by adopting an sIPTW-based analytic approach. Covariates that could be relevant but not available in the claims database and hence missing in our study are race/ethnicity, CD4 cell count, and HIV viral load. Adjustment for CD4 cell count and HIV viral load made little difference to the relative rate of AMI in a prior study [5]. There is potential for bias in the study results from residual confounding that may arise from the binary categorization of certain variables in the study, rather than having a graded continuous response. We assumed uninformative censoring for the study because participants in both the exposure groups, i.e., PLWH receiving abacavir based ART regimen and PLWH receiving non-abacavir based ART regimen, may be at similar risk of adverse HIV–related life events that may cease their continued enrollment into the health-plan and hence representation in the database. We chose AMI and/or coronary artery interventions only to define CVDe so as to be as specific as possible with the study outcome’s representation of ischemic CVD; however, we might use a broader definition including other cardiac conditions or cerebrovascular events for the study outcome.

## Conclusions

In summary, exposure to abacavir is associated with an increased risk of CVDe. We recommend a careful consideration of the risks and benefits of abacavir treatment while formulating antiretroviral treatment regimens with patients.

## Additional file

Additional file 1: Table S1. ICD-9-CM and CPT codes for defining various covariates and outcomes. **Table S2.** Age-specific incidence rate (IR) of acute myocardial infarction (AMI) among persons living with HIV receiving antiretroviral therapy. **Table S3.** Factors associated with initiation of abacavir among persons living with HIV, by pooled logistic regression. **Table S4** The influence of various risk factors on the development of CVD among persons living with HIV receiving anti-retroviral therapy. **Table S5** Risk of CVD from current exposure to abacavir in sub-groups of variables at baseline (test of interactions). **Table S6** Risk of cardiovascular disease from exposure to abacavir among persons living with HIV free of heart disease^a^ or substance or alcohol abuse at baseline. **Appendix 1.** Detailed approach to developing marginal structural models. (DOCX 58 kb)

## Abbreviations

AMI: Acute Myocardial Infarction
ART: Antiretroviral Therapy
ARV: Antiretroviral
CKD: Chronic Kidney Disease
CPT: Current Procedural Terminology
CVD: Cardiovascular Disease
CVDe: Cardiovascular Disease Events
D:A:D: Data Collection on Adverse Events of Anti-HIV Drugs
HIV: Human Immune Deficiency Virus
hsCRP: High Sensitivity C-Reactive Protein
ICD-9-CM: International Classification of Disease, 9th Revision, Clinical Modification
PLWHIV: People Living With HIV
sIPTW: Stabilized Inverse Probability Treatment Weights
