# Supplementary material for: Risk of cardiovascular events from current, recent, and cumulative exposure to abacavir among persons living with HIV who were receiving antiretroviral therapy in the United States: a cohort study
Source: BMC Infect Dis. 2017 Oct 27;17:708. doi: 10.1186/s12879-017-2808-8 (PMC5660446; doi:10.1186/s12879-017-2808-8)
Supplement: Additional file 1: Table S1. — ICD-9-CM and CPT codes for defining various covariates and outcomes. Table S2. Age-specific incidence rate (IR) of acute myocardial infarction (AMI) among persons living with HIV receiving antiretroviral therapy. Table S3. Factors associated with initiation of abacavir among persons living with HIV, by pooled logistic regression. Table S4 The influence of various risk factors on the development of CVD among persons living with HIV receiving anti-retroviral therapy. Table S5 Risk of CVD from current exposure to abacavir in sub-groups of variables at baseline (test of interactions). Table S6 Risk of cardiovascular disease from exposure to abacavir among persons living with HIV free of heart diseasea or substance or alcohol abuse at baseline. Appendix 1. Detailed approach to developing marginal structural models. (DOCX 58 kb) [file 12879_2017_2808_MOESM1_ESM.docx]

**Supporting Information**

**Additional Table 1. (Page 1)**

ICD-9-CM, and CPT codes for defining various covariates and outcomes.

**Additional Table 2. (Page 2)**

Age-specific incidence rate (IR) of acute myocardial infarction (AMI) among HIV-infected individuals receiving antiretroviral therapy.

**Additional Table 3. (Page 3)**

Factors associated with initiation of abacavir among HIV-infected individuals, by pooled logistic regression.

**Additional Table 4. (Page 4)**

The influence of various risk factors on the development of CVD among HIV-infected individuals receiving anti-retroviral therapy.

**Additional Table 5. (Page 5)**

Risk of CVD from current exposure to abacavir in sub-groups of variables at baseline (test of interactions).

**Additional Table 6. (Page 6)**

Risk of cardiovascular disease from exposure to abacavir among HIV-infected individuals free of heart disease or without a prior history of substance abuse at baseline.

**Appendix 1. (Page 7)**

Marginal Structural Model

**References (Page 9)**

**S1 Table. ICD-9-CM, and CPT codes for defining various covariates and outcomes**

| **Variable** | **ICD-9-CM Code** |
| --- | --- |
| Acute myocardial infarction | 410.xx |
| Percutaneous coronary intervention (CPT) | 92920-92921, 92924-92925, 92928-92929, 92933-92934, 92937-92938, 92941, 92943-92944, 92980-92981, 92984, 92996 |
| Coronary artery bypass graft (CPT) | 33510-33514, 33516-33519, 33521-33523, 33533-33536 |
| Tobacco use/Smoking | 305.1; v15.82 |
| Substance abuse (dependent and non-dependent) | 304.xx, 305.xx |
| Alcohol abuse (alcohol dependence and alcohol abuse) | 303.xx, 305.0x |
| Overweight/obese | 278.00, 278.01, 278.02 |
| Diabetes mellitus | 250.xx, 357.2x, 362.0x, 366.41 |
| Essential hypertension | 401.xx |
| Hypercholesterolemia | 272.0x |
| Hypertriglyceridemia | 272.1x |
| Mixed hyperlipidemia | 272.2x |
| Other and unspecified hyperlipidemia | 272.4x |
| Lipodystrophy | 272.6x |
| Chronic kidney disease | 585.xx |
| Heart failure | 402.01, 402.91, 428%, 404.01, 404.03,404.11,404.13,404.91, 404.93 |
| Cardiac dysrhythmia | 427.xx |
| Old myocardial infarction | 427.xx |
| Coronary atherosclerosis | 414.xx |
| Stroke | 434.xx |
| Hepatitis B virus infection | 070.2x, 070.3x, V02.61 |
| Hepatitis C virus infection | 070.41, 070.44, 070.51, 070.54, 070.7x, v02.62 |
| Any cancer | 140-149, 150-159, 160-169, 170-179, 180-189, 190-199, 200-209, 210-229, 230-239 |

ICD-9-CM: International Classification of Disease, 9^th^ Revision, Clinical Modification; CPT: Current Procedural Terminology

**S2 Table. Age-specific incidence rate (IR) of acute myocardial infarction**

**(AMI) among HIV-infected individuals receiving antiretroviral therapy**

| **Age-group** | **Person-years** | **Number of AMI** | **IR per 1000 people**  **(95% CI)** |
| --- | --- | --- | --- |
| 18-39 | 27,869 | 33 | 1·18 (0·84, 1·67) |
| 40-49 | 46,677 | 149 | 3·19 (2·72, 3·75) |
| 50-59 | 32,852 | 259 | 7·88 (6·98, 8·90) |
| 60-69 | 6,779 | 97 | 14·31 (11·73, 17·46) |
| >=70 | 562 | 10 | 17·80 (9·58, 33·08) |
| Overall | 114,738 | 548 | 4·78 (4·39, 5·19) |

**S3 Table. Factors associated with initiation of abacavir among HIV-infected individuals, by pooled logistic regression (Treatment model).**

| **Variable** | **Hazard Ratio (95% CI)** | **P value** |
| --- | --- | --- |
| Male sex^a^ | 0·90 (0·86, 0·96) | <0·001 |
| Age^b^ | 1·03 (1·029, 1·034) | <0·001 |
| Calendar year^b^  2009  2010  2011  2012  2013  2014 | Reference  0·84 (0·78, 0·90)  0·80 (0·75, 0·86)  0·70 (0·65, 0·75)  0·63 (0·58, 0·68)  0·68 (0·63, 0·73) | <0·001  <0·001  <0·001  <0·001  <0·001 |
| Ever tobacco use/smoking | 1·03 (0·94, 1·14) | 0·50 |
| Ever substance/alcohol abuse | 0·83 (0·76, 0·91) | <0·001 |
| Symptomatic HIV infection^a^ | 1·09 (1·03, 1·15) | 0·002 |
| Any diagnosis of cancer^a^ | 0·85 (0·78, 0·94) | 0·001 |
| Diagnosis of chronic kidney disease^b^ | 4·22 (3·73, 4·77) | <0·001 |
| Receipt of medications for  heart disease^bc^ | 0·89 (0·83, 0·95) | 0·001 |
| History of lipodystrophy^b^ | 1·75 (1·37, 2·24) | <0·001 |
| Diagnosis of dyslipidemia^b^ | 1·03 (0·96, 1·11) | 0·40 |
| Prior AMI^bd^ | 1·21 (0·77, 1·88) | 0·41 |
| History of heart failure, cardiac arrhythmia or atherosclerosis^b^ | 0·92 (0·81, 1·04) | 0·17 |
| Diagnosis of hypertension^b^ | 1·06 (0·98, 1·16) | 0·13 |
| Diagnosis of diabetes mellitus or receipt of anti-hyperglycemic agents^b^ | 0·99 (0·90, 1·10) | 0·91 |
| Hepatitis B^a^ | 0·87 (0·69, 1·10) | 0·25 |
| Hepatitis C^a^ | 1·16 (0·98, 1·37) | 0·08 |
| BMI>24.9^b^ | 0·95 (0·81, 1·11) | 0·50 |
| History of stroke^a^ | 0.91 (0.61, 1.37) | 0.66 |

^a^ Baseline variables.

^b^ Time-dependent variables. The first observation of a time-dependent covariate corresponds

to its baseline value.

^c^ aspirin, beta-blocker, statins, angiotensin converting enzyme inhibitor,

angiotensin receptor blocker, calcium channel blocker.

^d^ acute myocardial infarction.

**S4 Table**. **The influence of various risk factors on the development of CVD among HIV-infected individuals receiving anti-retroviral therapy**

| **Variable** | **Adjusted Cox Model^c^**  **HR (95% CI)** | **P-value** |
| --- | --- | --- |
| Age (per year)^b^ | 1.06 (1.05, 1.07) | <0.001 |
| Male sex^a^ | 1.83 (1.42, 2.36) | <0.001 |
| Tobacco use/smoking (ever) | 1.53 (1.19, 1.98) | <0.001 |
| Substance/alcohol abuse (ever) | 1.10 (0.85, 1.42) | 0.46 |
| Calendar year^b^  2009  2010  2011  2012  2013  2014 | Referent  0.92 (0.61, 1.38)  0.95 (0.63, 1.44)  0.80 (0.52, 1.22)  0.74 (0.48, 1.13)  0.91 (0.59, 1.39) | -  0.69  0.81  0.29  0.16  0.65 |
| BMI>24.9^b^ | 0.86 (0.62, 1.19) | 0.37 |
| Symptomatic HIV infection^a^ | 0.89 (0.74, 1.07) | 0.21 |
| Heart failure, cardiac dysrhythmia, and atherosclerosis^b^ | 4.13 (3.45, 4.95) | <0.001 |
| Old myocardial infarction^a^ | 3.38 (2.02, 1.75) | <0.001 |
| Use of medications for  heart disease^b^ | 1.46 (1.22, 1.75) | <0.001 |
| Diabetes Mellitus^b^ | 1.32 (1.09, 1.60) | 0.005 |
| Essential Hypertension^b^ | 1.17 (0.97, 1.42) | 0.10 |
| Dyslipidemia^b^ | 1.35 (1.13, 1.61) | 0.001 |
| Lipodystrophy^b^ | 1.24 (0.86, 1.79) | 0.25 |
| Chronic Kidney Disease^b^ | 1.04 (0.79, 1.36) | 0.79 |
| Hepatitis B^a^ | 0.79 (0.32, 1.96) | 0.62 |
| Hepatitis C^a^ | 1.61 (0.99, 2.64) | 0.06 |
| Cancer^a^ (any) | 0.77 (0.55, 1.07) | 0.19 |

^a^ baseline variables; ^b^ time dependent variables

^c^ Cox proportional hazard model adjusted for baseline covariates: gender, tobacco use (ever), substances or alcohol abuse (ever), symptomatic HIV disease, serologic evidence of hepatitis B & C infections, history of stroke, history of cancer, prior myocardial infarction, and time-dependent covariates: age, calendar year, body weight, receipt of anti-hyperglycemic agents, receipt of medications for heart disease, and diagnoses of: diabetes mellitus, chronic kidney disease, dyslipidemia, heart failure, cardiac dysrhythmia, atherosclerosis, and hypertension.

**S5 Table**. **Risk of CVD from current exposure to abacavir in**

**sub-groups of variables at baseline (test of interactions)**

| **Variable** | **^a^Hazard Ratio**  **(95% CI)** | **P-value for test of interaction** |
| --- | --- | --- |
| Age>45  Age<=45 | 1.43 (1.16, 1.76)  2.55 (1.59, 4.09) | 0.03 |
| Female  Male | 1.00 (0.49, 2.04)  1.37 (1.25, 1.85) | 0.27 |
| Chronic Kidney Disease (CKD)  No CKD | 1.08 (0.43, 2.74)  1.47 (1.21, 1.78) | 0.53 |
| ^b^Heart disease  No heart disease | 0.65 (0.33, 1.30)  1.58 (1.30, 1.93) | 0.02 |
| Medications for heart disease  No medications for heart disease | 1.13 (0.68, 1.88)  1.53 (1.25, 1.88) | 0.27 |
| Dyslipidemia  No dyslipidemia | 1.24 (0.71, 2.09)  1.51 (1.23, 1.85) | 0.46 |
| Diabetes mellitus  No diabetes mellitus | 1.18 (0.59, 2.35)  1.49 (1.27, 2.47) | 0.53 |
| Lipodystrophy  No lipodystrophy | 5.24 (0.46, 60.13)  1.32 (1.09, 1.60) | 0.27 |
| Hypertension  No Hypertension | 1.45 (0.86, 2.43)  1.47 (1.20, 1.80) | 0.96 |
| Substance abuse  No substance abuse | 1.61 (0.63, 4.10)  1.46 (1.21, 1.78) | 0.85 |
| Alcohol abuse  No alcohol abuse | 3.30 (0.71, 15.34)  1.46 (1.20, 1.76) | 0.30 |
| Overweight/obese  Not overweight/obese | 0.78 (0.10, 6.28)  1.48 (1.22, 1.79) | 0.55 |
| Smoker  Non smoker | 2.66 (1.11,6.40)  1.43 (1.18, 1.74) | 0.17 |

^a^ A separate pooled logistic model containing the corresponding interaction term was run for each

variable after adjusting for a uniform set of covariates as defined previously for other models.

^b^ Includes individuals receiving medications for cardiovascular disease.

**S6 Table. Risk of cardiovascular disease from a current exposure to abacavir among HIV-infected individuals free of heart disease**^a^ **or without a prior history of substance abuse at baseline.**

| **Risk group** | **Unadjusted Cox Model**  **HR (95% CI; p value)** | **Adjusted Cox Model**  **HR^b^ (95% CI; p value)** | **Marginal Structural Model**  **HR^c^ (95% CI; p value)** |
| --- | --- | --- | --- |
| Individuals free of heart disease at baseline ^d^ | 1.82 (1.50, 2.22; p<0.001) | 1.41 (1.16, 1.72; p=0.001) | 1.53 (1.26, 1.87; p<0.001) |
| Individuals without a prior history of substance or alcohol abuse ^e^ | 1.69 (1.40, 2.04; p<0.001) | 1.31 (1.08, 1.58; p=0.007) | 1.41 (1.16, 1.71; p=0.001) |

^a^ Includes heart failure, cardiac dysrhythmia, atherosclerosis, and prior myocardial infarction.

^b^ Adjusted for baseline covariates: gender, tobacco use (ever), symptomatic HIV disease, serologic evidence of hepatitis B & C infections, history of stroke, history of cancer and time-dependent covariates: age, calendar year, body weight, receipt of anti-hyperglycemic agents, receipt of medications for heart disease, and diagnoses of: diabetes mellitus, chronic kidney disease, dyslipidemia, heart failure, cardiac dysrhythmia, atherosclerosis, and hypertension.

^c^ In addition to adjusting for weights generated from the treatment model using the time-fixed and time-dependent covariates in the adjusted Cox model, the marginal model is adjusted for time-fixed/baseline covariates: sex, ever tobacco use, symptomatic HIV disease, serologic evidence of hepatitis B & C infections, history of stroke, history of cancer, and baseline values of time-dependent covariates: age, calendar year, receipt of anti-hyperglycemic agents, receipt of medications for heart disease, and diagnoses of: diabetes mellitus, chronic kidney disease, and dyslipidemia.

^d^Treatment and marginal model are additionally adjusted for substance abuse

^e^Treatment and marginal models are additionally adjusted for heart disease

**S1 Appendix.**

**Marginal Structural Model**

**Rationale and Definition:**

Because the estimates generated by traditional methods could be biased in the presence of confounders that lie on the causal pathway between the exposure and the outcome, and were also predicted by past exposure to the ARV agent of interest, we used a causal inference approach that estimates the parameters of marginal structural models using inverse probability weights, which can account for the issue of time-dependent confounding^1^. In this study, the relationship between abacavir use and risk of CVD may be confounded by covariates such as diabetes mellitus, hypertension, dyslipidemia, lipodystrophy, and chronic kidney disease, and the values of these covariates could be influenced by past exposure to abacavir or the comparator ARV agents, such as PIs. The specified MSM will model the hazard of AMI had everyone in the study population received the ARV agent of interest compared to if everyone had not received the exposure. In the context of time-dependent covariates, the effect estimate from the usual Cox proportional hazards model is an unbiased estimate of the associational parameter but it is a biased estimator of the causal effect of the specified exposure on survival among HIV-infected individuals receiving ART. Therefore, we employed the marginal structural Cox proportional hazards method (Hernan et al, 2000)^2^. Adopting the notations used by Hernan et al, we defined $T$to be the patient’s time to cardiovascular disease events (CVDe) or the censoring date, with time measured in months, and $A\left( t \right)=1$if the subject received the specified exposure at a given time t, where $0\leq u\leq t$. $\bar{A}\left( t \right)$ represents patient’s treatment history up to time t. $V$ represents the vector of time-independent baseline covariates. $L(t)$ represents the vector of time-dependent covariates at time$t, \mathrm{and}T_{\bar{a}}$ represents the counterfactual random variable that represents patient’s time to outcome, had he/she experienced the exposure history from the start of follow up rather than his/her observed history. We observe $T_{\bar{a}}$ only for those patients’ exposure histories $\bar{a,}$ where the subject actually received the exposure, in our case, the specified ARV agent, from start of follow up until the development of CVDe or the censor date. Then $T_{\bar{a}}$ equals $T,$and for each $\bar{a}$, an example of a marginal structural cox proportional hazard model is given by:

$\lambda_{T_{\bar{a}}}\left( t | V \right)=\lambda_{0}\left( t \right) exp(\beta_{1}a\left( t \right)+\beta_{2}V)$ (1)

where $\lambda_{T_{\bar{a}}}\left( t | V \right)$ was the hazard of CVDe among subjects with baseline covariates $V$ had, contrary to fact, all subjects followed the specified exposure history through $\bar{a}$. This model is a marginal structural model as it assumes a smooth (parametric) function relating the subset of covariates and counterfactual levels of treatment to the hazard. We obtained the parameters of MSM defined in (1) by fitting a pooled logistic regression that essentially models the hazard of CVDe,

$\lambda_{T}\left( t | \bar{A}\left( t \right), V \right)=\lambda_{0}\left( t \right)exp(\beta_{1}^{*}A\left( t \right)+\beta_{2}^{*}V)$ (2)

using a stabilized weight

${sw}_{i}=\prod_{k=0}^{int\left( t \right)} \frac{pr\left( A\left( k \right)=a_{i}\left( k \right)|\bar{A}\left( k-1 \right)=\bar{a}_{i}(k-1), V=v_{i} \right)}{pr\left( A\left( k \right)=a_{i}\left( k \right)|\bar{A}\left( k-1 \right)=\bar{a}_{i}(k-1), V=v_{i}, \bar{L}\left( k \right)=\bar{l}_{i}\left( k \right) \right)}$ (3)

where $\bar{A}(-1)$ is defined to be 0 and $int(t)$ is the largest integer less than or equal to $t$ and $k$ denotes months since start of follow up. The marginal structural model was adjusted for the sIPTW and the baseline covariates. The same treatment weights were used for estimation of CVDe risk from current, recent, and cumulative exposure to abacavir.

**Estimation of the Weights**

Assuming sufficiency of the measured time dependent covariates for identifiability of the causal question of ART use and risk of AMI/CVD [sequential randomization assumption, ^3^], the use of stabilized weights sw­_i_ effectively generates, in a risk set at time t, a pseudo-population in which $\bar{L}(t)$ no longer predicts the specified exposure use and hence $\bar{L}(t)$ is no longer a confounder^1^ and the causal parameter would be same as that in the original study population^1^. The stabilized weight was generated using a pooled logistic regression as follows, based upon the methods described by Cole et al^4^. Same weight generating model as for those receiving abacavir continuously was used for patients who restarted abacavir after stopping for more than 30 days.

**Model for numerator of sIPTW:**

Numerator: The numerator of the stabilized weight was modelled using a logistic model

${Logit pr(A_{k}=1|\bar{A}}_{k-1}={\bar{a}_{k-1},V})=\alpha_{0}^{*}+{\alpha_{1}^{*}a_{k-1}+\alpha}_{2}^{*}V+ \alpha_{3}^{*}k$ (4)

where the probability of current treatment was allowed to be a function of the last month’s treatment history, baseline/time-fixed covariates, and time modelled as a smoothed function of natural cubic splines with three internal knots at 25^th^, 50^th^, and 75^th^ percentile corresponding to month 6, month 14, and month 26.

**Model for denominator of sIPTW:**

Denominator: The denominator of the stabilized weight was modelled using the logistic model

${Logit pr(A_{k}=a_{k}|\bar{A}}_{k-1}={\bar{a}_{k-1},\bar{L}_{k}=\bar{l}_{k}, V})=\alpha_{0}+\alpha_{1}a_{k-1}+\alpha_{2}l_{k}+{\alpha_{3}V+\alpha}_{4}k$ (5)

where the probability of receiving current treatment depended upon the last month’s treatment history, time-dependent and time-fixed covariates, and time modelled as a function of natural cubic splines as that of the numerator. We then obtained the predicted probabilities of receiving treatment for each individual from the logistic models of (4) and (5), which we then used to calculate the stabilized weight as

${sw}_{i}=\frac{\prod_{k=0}^{K} \left( \hat{p}_{ki}^{*} \right)^{a_{ki}}\left( 1-\hat{p}_{ki}^{*} \right)^{\left( {1-a}_{ki} \right)}}{\left\{ \prod_{k=0}^{K} \left( \hat{p}_{ki} \right)^{a_{ki}}\left( 1-\hat{p}_{ki} \right)^{\left( {1-a}_{ki} \right)} \right\}}$ (6)

Hence, by using the stabilized weights, we obtained an unbiased estimate of the causal parameter β­_1_. $e^{\beta_{1}}$ is the causal hazard ratio for the effect estimate of the hazard of CVDe in HIV-infected individuals receiving the specified exposure, compared to HIV-infected individuals receiving the reference exposure. The sIPTWs help to create a pseudo-population in which the treatment is not confounded by the covariates, and the causal parameters generated from the pseudo-population are the same as those of the true population (Robins et al., 2000). Robins et al. also showed that the stabilized weights have smaller variance as compared to non-stabilized IPTW weights, leading to narrower confidence intervals. We also assessed effect modification by including interaction terms between time independent baseline covariates and the treatment variable in the MSM. The 95% confidence interval was calculated using the Huber White robust or sandwich estimator of the variance for β1 as $\hat{\beta}_{1}$±1.96$\sqrt{var\left( \hat{\beta}_{1} \right)}$.

**References.**

1. Robins JM, Hernan MA, Brumback B. Marginal structural models and causal inference in epidemiology. *Epidemiology.* 2000;11(5):550-560.

2. Hernan MA, Brumback B, Robins JM. Marginal structural models to estimate the causal effect of zidovudine on the survival of HIV-positive men. *Epidemiology.* 2000;11(5):561-570.

3. Bryan J, Yu Z, Van Der Laan MJ. Analysis of longitudinal marginal structural models. *Biostatistics.* 2004;5(3):361-380.

4. Cole SR, Hernan MA. Constructing inverse probability weights for marginal structural models. *Am. J. Epidemiol.* 2008;168(6):656-664.
